# Coffee inhibition of CYP3A4 in vitro was not translated to a grapefruit‐like pharmacokinetic interaction clinically

**DOI:** 10.1002/prp2.346

**Published:** 2017-09-27

**Authors:** George K. Dresser, Brad L. Urquhart, Julianne Proniuk, Alvin Tieu, David J. Freeman, John Malcolm Arnold, David G. Bailey

**Affiliations:** ^1^ Lawson Health Research Institute London Health Sciences Centre Western University London Ontario Canada; ^2^ Department of Medicine Schulich School of Medicine & Dentistry Western University London Ontario Canada; ^3^ Department of Physiology & Pharmacology Schulich School of Medicine & Dentistry Western University London Ontario Canada

**Keywords:** Coffee, drug metabolism, grapefruit, pharmacokinetics

## Abstract

Grapefruit can augment oral medication bioavailability through irreversible (mechanism‐based) inhibition of intestinal CYP3A4. Supplementary data from our recent coffee–drug interaction clinical study showed some subjects had higher area under the plasma drug concentration ‐ time curve (AUC) and plasma peak drug concentration (Cmax) of the CYP3A4 probe felodipine compared to aqueous control. It was hypothesized that coffee might interact like grapefruit in responsive individuals. Beans from six geographical locations were consistently brewed into coffee that was separated chromatographically to a methanolic fraction for in vitro inhibition testing of CYP3A4 metabolism of felodipine at 1% coffee strength. The effect of simultaneous incubation and 10‐min preincubation with coffee fractions determined whether coffee had direct and mechanism‐based inhibitory activity. A subsequent five‐way randomized balanced controlled crossover clinical study evaluated the clinical pharmacokinetic interaction with single‐dose felodipine. Grapefruit juice, water, or three of the in vitro tested coffees were ingested at 300 mL alone 1 h before and then with felodipine. In vitro, all six coffees decreased felodipine metabolism for both simultaneous and preincubation exposure compared to corresponding control. Five coffees demonstrated mechanism‐based inhibition. Grapefruit increased felodipine AUC_0–8_ (25 vs. 13 ng.h/mL, *P* < 0.001) and Cmax (5.8 vs. 2.7 ng/mL, *P* < 0.001) and decreased dehydrofelodipine/felodipine AUC_0–8_ ratio (0.84 vs. 1.29, *P* < 0.001), while the three coffees caused no change in these parameters compared to water. Despite high in vitro potency of CYP3A4 inhibition, the coffees did not cause a clinical pharmacokinetic interaction possibly from insufficient amount of inhibitor(s) in coffee reaching intestinal CYP3A4 during the absorption phase of felodipine. The results of this study highlight the need for follow‐up clinical testing when in vitro results indicate the possibility of an interaction.

## Introduction

Food–drug interactions can be therapeutically challenging. Grapefruit and other fruit juices have been identified to alter drug disposition through a couple of different mechanisms. Reduced oral drug bioavailability can occur for nonmetabolized hydrophilic drugs that require intestinal uptake transport through inhibition of the action of certain organic anion transporting polypeptides like OATP1A2 or OATP2B1 (Dresser et al. 2002; Bailey 2010). On the other hand, augmented oral drug bioavailability can happen for lipophilic drugs that normally undergo substantial intestinal metabolism through inhibition of CYP3A4 (Bailey et al. 1991, 2013). The latter interaction is the focus of this study and it has been documented or predicted to occur for more than 85 drugs (Bailey et al. 2013). The clinical relevance of this outcome is contingent upon the magnitude of increase of systemic drug concentration in the context of the seriousness of overdose toxicity. The primary clinical issue is the risk of greater frequency of adverse drug events.

The basis for grapefruit–drug interactions was irreversible inactivation (mechanism‐based inhibition) of a crucial human‐specific drug metabolizing enzyme, CYP3A4, particularly in the small intestines (Lown et al. 1997). The magnitude of effect was individual dependent (Bailey et al. 1995). Higher intestinal CYP3A4 content was associated with greater increase in the plasma drug concentration (Lown et al.^1997^).

Citrus fruits related to grapefruit like Seville orange, pomelo, and lime also caused this interaction clinically and contained the same furanocoumarins that potently inhibited in vitro CYP3A4 activity (Malhotra et al. 2001; Bailey et al. 2003; Guo and Yamazoe 2004; Guo et al. 2007). Theoretically, other distinctly different foods and constituents might have these properties. Our recent study showed that coffee induced an acute pressor effect after a 2‐day abstinence that partially blocked the antihypertensive action of felodipine through a pharmacodynamic interaction (Bailey et al. 2016). Supplementary data showed that 4 of the 13 study subjects had felodipine AUC and Cmax with coffee that ranged 155–257% and 165–367% of those with aqueous control, respectively. Felodipine was one of the first drugs reported to interact with grapefruit and has been used in subsequent investigations as a probe for this effect (Bailey et al. 1989, 1991). We hypothesized that coffee might act analogously to grapefruit.

Coffee is much more commonly consumed than grapefruit juice and thus might have a greater chance of being ingested with grapefruit‐affected drugs during therapy. Beans of the plant, *Coffea arabica*, constitute 80% of the world trade. Because of the importance of growing conditions in determining the aroma and taste of coffee, the beans are named for their geographical origin. Roasting, grinding, and brewing conditions also affect the characteristics of the final product and the constituents in coffee that might play a role in this interaction.

This is the first report to our knowledge to assess the effect of coffee on drug metabolism and pharmacokinetics. Coffee beans from six geographical locations that differed in the extent of roasting were consistently ground and brewed. The methanol eluent chromatographic fraction of each freshly brewed coffee equivalent to 1% normal strength was tested for in vitro inhibition of felodipine metabolism. Three of these coffees differing in mechanism‐based potency and roasting were then evaluated in a clinical interaction study in five healthy volunteers.

## Materials and Methods

### In vitro inhibition of CYP3A4 activity

#### Preparation of coffee and extract

Coffee beans were purchased from a local outlet of Starbucks Coffee Canada Inc (North York, Ontario, M2N 6L7). They differed in geographical origin (extent of roasting) and included Breakfast Blend from Latin America (mild), Guatemala (medium), Organic Mexico (medium), Kenya (bold), Red East Africa (bold), and Sumatra (extra bold). Beans were ground to medium coarseness and three tablespoons per 300 mL cold double‐distilled water were brewed in a Starbucks Barista Aroma Grand Thermal Coffee Maker. Following cooling to room temperature, 1000 μL of coffee was applied to a 100‐mg C18 preparatory solid phase extraction column (Sep‐Pak Vac cartridge; Waters Corp, Mississauga, Ontario, Canada), which had been previously washed with 1 ml volumes of isopropyl alcohol, methanol, and water. It was eluted with 1000 μL methanol to obtain the concentrated extract for CYP3A4 inhibition studies.

#### Preparation of CYP3A4 solution

A 500‐μL vial of CYP3A4 containing cytochrome P450 reductase and cytochrome b_5_ shipped in dry ice (Sigma‐Aldrich Chemical Co., St Louis, MO, USA) was stored at −80°C immediately upon receipt. It was later gradually thawed in ice and diluted with cold phosphate buffer 100 mmol/L pH 7.4–7.5 to obtain CYP3A4 2 pmol/100 μL. Then, 500 μL aliquots were transferred to microcentrifuge tubes prechilled in ice and stored at −80°C. They were thawed in ice just before testing as needed.

#### Simultaneous exposure of coffee extracts to CYP3A4

The negative control initially equilibrated 100 μL of CYP3A4 solution, 4 μL of felodipine 1000 ng/mL in methanol (final concentration 20 ng/mL), and 2 μL of methanol for 10 min at 38.5°C. The positive control had 2 μL of bergamottin 10 μg/mL in methanol (final concentration 0.10 μg/mL) instead of the 2 μL of methanol in the negative control. The coffee treatment had 2 μL of coffee extract (final concentration 1% of originally brewed coffee) to replace the 2 μL of methanol. Then, 100 μL of NADPH 0.3 mmol/L in phosphate buffer was added and the mixture was incubated for a further 15 min at 38.5°C. The reaction was terminated by the addition of 200 μL of toluene containing the internal standard (H165/04; AB Haessle, Gothenburg, Sweden) at 20 ng/mL that was vortexed for 15 sec and then centrifuged. The aqueous phase was frozen at −20°C and the toluene phase was removed for analysis of the felodipine concentration. Results were the mean of triplicate in vitro inhibition assays.

#### Prior exposure of coffee extracts to CYP3A4

The procedure was the same as that for simultaneous exposure but with a few modifications. The negative control initially equilibrated 100 μL of CYP3A4 solution, 100 μL of NADPH 0.3 mmol/L in phosphate buffer, and 2 μL of methanol for 10 min at 38.5°C. The positive control had 2 μL of bergamottin 10 μg/mL in methanol (final concentration 0.10 μg/mL) and the coffee treatment had 2 μL of the extract (final concentration 1% of originally brewed coffee) instead of the 2 μL of methanol in the negative control. Then, 4 μL of felodipine 1000 ng/mL in methanol (final concentration 20 ng/mL) was added for incubation for 15 min at 38.5°C.

#### Assay of felodipine and dehydrofelodipine

Samples were analyzed based on a previous method with modifications (Ahnoff 1984). Preparation of samples for injection are outlined for the in vitro investigation (above) and for the clinical study (below). Briefly, 1 μL of the toluene extract was introduced by splitless injection into a dual‐tapered deactivated glass insert (Hewlett Packard Canada Ltd., Toronto, Ontario, Canada) to prevent chemical oxidation of felodipine in the injector port. Chromatography was performed on a Hewlett Packard 5890 Series II Gas Chromatograph (Hewlett Packard Canada Ltd) equipped with a ^63^Ni electron capture detector and a 25‐m × 0.32‐mm inner diameter fused silica capillary column coated with a stationary phase of methyl silicone, 0.52‐μm (HP‐1; Hewlett Packard Canada Ltd.). After a purge for 1 min, the initial oven temperature of 90°C was increased at 30°C/min to 180°C, at 5°C/min to 260°C for 3 min, and then at 30°C/min to a final temperature of 280°C for 5 min. The injector port and detector temperatures were maintained at 260°C and 300°C, respectively. The carrier gas was ultrapure helium (column inlet pressure of 100 kPa), and the make‐up gas was ultrapure nitrogen (60 mL/min). Retention times of felodipine, dehydrofelodipine, and internal standard were 20.1, 14.5, and 21.7 min, respectively. The coefficients of variation for felodipine and dehydrofelodipine concentrations were 4.7% and 2.9% at 1.0 ng/mL (*n* = 5). The limit of detection was 0.25 ng/mL for both.

#### Data analyses

One‐way ANOVA was initially used with each of the six coffees for simultaneous and prior exposure which resulted in *P* < 0.05 in every case. This was followed by Tukey's multiple comparison test for all pairs (15 sets).

The magnitude of the mechanism‐based inhibition was calculated as the simultaneous minus 10‐min prior exposure difference in metabolized felodipine concentration between the same treatment within each coffee.

### Clinical interaction study

#### Study population

Five study subjects (3 men and 2 women; mean age 46 [range, 31–56 years]) were healthy as determined by medical history, physical examination, and routine hematologic and serum chemical analysis. They had no significant illness within the preceding 2 weeks, received no investigational drug within the previous 4 weeks, had no history of cardiac, renal, hepatic, gastrointestinal disease, or drug/alcohol abuse. The University of Western Ontario Research Ethics Board for Health Sciences Research Involving Human Subjects approved the study. All subjects provided written informed consent and completed the study.

#### Experimental protocol

This was a five‐way crossover, controlled (positive and negative), balanced, randomized, open‐labeled, single‐dose oral pharmacokinetic investigation. The treatments were: (1–3) a black coffee plus felodipine, (4) grapefruit juice plus felodipine, and (5) water plus felodipine. The selected three coffees had different in vitro mechanism‐based inhibitory potency and extent of roasting (mild, medium, extra bold). Volunteers were tested on five study days separated by an interval of 1 week. They were asked to avoid taking grapefruit, Seville orange (marmalades), lime, pomelo, coffee, other caffeine‐containing beverages, tobacco, alcoholic drinks, medications (prescription or over‐the‐counter), and natural health products for at least 48 h before each study day. Testing was preceded by a 10‐h overnight fast. On each study day, a baseline plasma sample was taken to measure caffeine concentration that was used as a surrogate marker of coffee consumption during the run‐in period. Volunteers then received freshly brewed coffee (see preparation above), grapefruit juice made from 100% frozen concentrate (Minute Maid Company Canada, Inc., Toronto, Canada) or water, which were taken as 300 mL alone (study hour −1) and then 300 mL with felodipine (study hour 0). Felodipine 10 mg was given as the extended‐release tablet (Plendil; Astra Pharma, Inc., Mississauga, Canada). Plasma felodipine, dehydrofelodipine (primary metabolite formed by CYP3A4), and caffeine concentrations were measured at specific time points over the 8 h of study. For felodipine, plasma (500 μL) was extracted with toluene (250 μL) containing the internal standard (H165/04; AB Haessle, Gothenburg, Sweden) by gently oscillating the mixture overnight. After centrifugation and freezing of the aqueous phase at −20°C, the supernatant toluene phase was removed for analysis as outlined above. Blood pressure was monitored for safety. Adverse events reported by open questioning, volunteered by the subject or observed by the clinic staff were documented. A lunch consisting of a sandwich, ginger ale, and ice cream sandwich was provided 4 h after drug dosing (noon). Tobacco and other caffeine‐containing beverages were not permitted during testing.

#### Assay of caffeine

Plasma samples were quantified for caffeine based on an earlier procedure (Carey and DePalma 1994). The method employed solid phase extraction followed by analysis on a Waters H‐class ultraperformance liquid chromatography (UPLC) system coupled to a Waters photodiode array detector. Briefly, plasma (200 μL) was diluted with water (800 μL) and internal standard (7‐*β*‐hydroxypropyl theophylline, 50 μg/mL) solution (50 μL). The samples were then passed across Strata‐X solid phase extraction cartridges (Phenomenex, Torrance, CA, USA) followed by water (2 × 1 mL) and 20% methanol/water wash solution (1 mL). The analytes were eluted into clean dry glass test tubes with methanol (1 mL) containing 0.1% triethylamine and 0.1% trifluoroacetic acid and evaporated to dryness. The residue was reconstituted in 0.1% acetic acid in water (200 μL). Analytes were separated on a Phenomenex Kinetex XB‐C18 (50 × 2.1 mm, 1.7 μm) column maintained at 40°C using an isocratic mobile phase consisting of 5 mmol/L KH_2_PO_4_ with 0.1% triethylamine (pH = 4.0) and acetonitrile in a ratio of 90:10. The column was washed with 80% acetonitrile for 1 min and re‐equilibrated at initial conditions for one minute prior to the next injection. Caffeine and 7‐*β*‐hydroxypropyl theophylline were eluted with retention times of 0.64 and 0.82 min, respectively, and were well resolved from matrix components and other contaminants. Caffeine and internal standard 7‐*β*‐hydroxypropyl theophylline were monitored at 270 nm. The coefficient of variation of the assay was 5.8% with a limit of accurate quantitation of 0.2 μg/mL.

#### Data analyses

The area under the plasma drug concentration–time curve (AUC) was calculated from 0 to 8 h by the linear trapezoidal method. Plasma peak drug concentrations (Cmax) and the time to reach Cmax (tmax) were obtained directly from the experimental data. The terminal elimination rate constant (ke) was determined by log‐linear regression of the final data points (at least three). The apparent elimination half‐life of the log‐linear phase (*t*1⁄2) was calculated as 0.693/ke.

Since one of the five subjects was not a responder with grapefruit juice (felodipine AUC and Cmax were 107% and 130% of those with water, respectively), intention to treat and per protocol analyses were conducted. This consisted of ANOVA for repeated measures initially for comparisons among the groups. For those analyses with *P* < 0.05, Bonferroni test for multiple comparisons was subsequently conducted between selected pairs of treatments (felodipine with water versus other four treatments). Statistical differences were the same between the two types of analyses and only intention to treat data are presented. Results are presented as the mean ± SEM.

## Results

### In vitro inhibition of CYP3A4 activity

**Figure 1:**
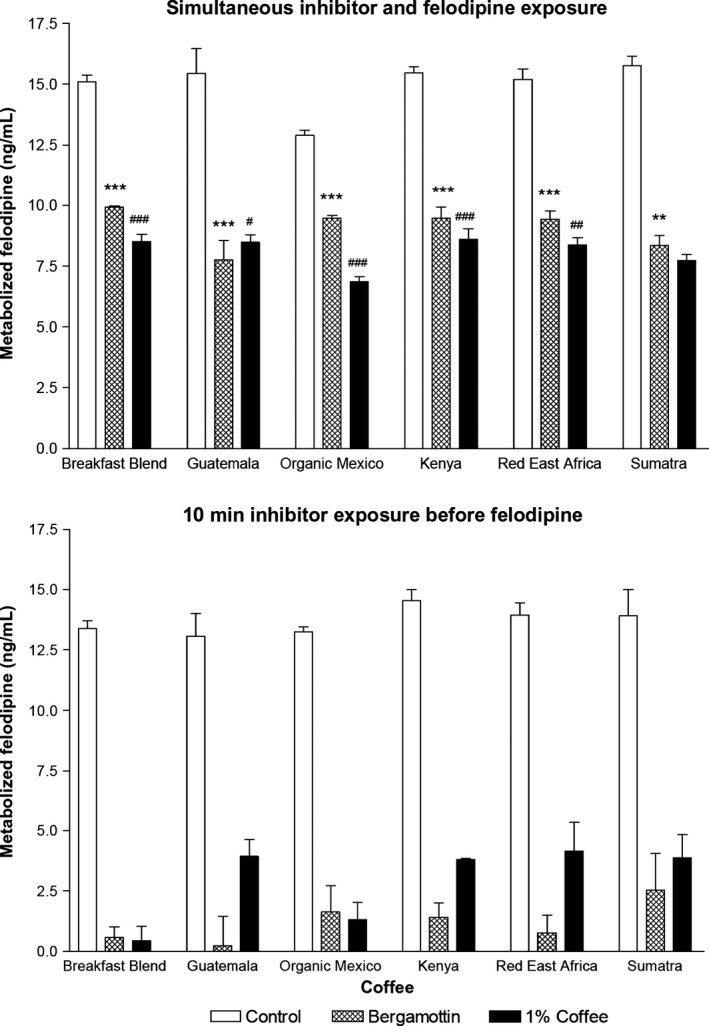
Felodipine metabolized by CYP3A4 for negative control, mechanism‐based inhibitor bergamottin, and 1% strength fraction from six coffees. One method was simultaneous incubation of bergamottin or coffee fraction and felodipine with CYP3A4 (upper bar graph). The other was a 10‐min prior incubation of bergamottin or coffee fraction with CYP3A4 before addition of felodipine (lower bar graph). Data (*n* = 3) are the mean ± SEM. **P < 0.01, ***P < 0.001 for comparisons between bergamottin values for simultaneous and prior incubation methods for each coffee. ^#^ *P*<0.05, ^#^^#^ *P* < 0.01, ^#^^#^^#^ *P* < 0.001 for comparisons between coffee fraction values for simultaneous and prior incubation methods for each coffee.

Bergamottin and 1% Breakfast Blend, Guatemala, Organic Mexico, Kenya, Red East Africa, and Sumatra coffee fractions had less felodipine metabolism compared to their corresponding control for both simultaneous and prior exposure (range: *P* < 0.01–*P* < 0.001, Fig. 1). Since the two controls were not different between testing methods of each coffee (*P* > 0.05), the two other matching treatments were compared. Bergamottin had more felodipine metabolism for simultaneous compared to prior exposure for each coffee (range: *P* < 0.01–*P* < 0.001). The 1% coffee fraction had the same for Breakfast Blend (mean: 8.5 vs. 0.4, *P* < 0.001), Guatemala (8.5 vs. 3.9, *P* < 0.05), Organic Mexico (6.9 vs. 1.3, *P* < 0.001), Kenya (8.6 vs. 3.8, *P* < 0.001), and Red East Africa (8.4 vs. 4.2, *P* < 0.01) coffees. However, it was not different for Sumatra (7.7 vs. 3.9, *P* > 0.05).

### Clinical interaction study

#### Felodipine pharmacokinetics

**Figure 2:**
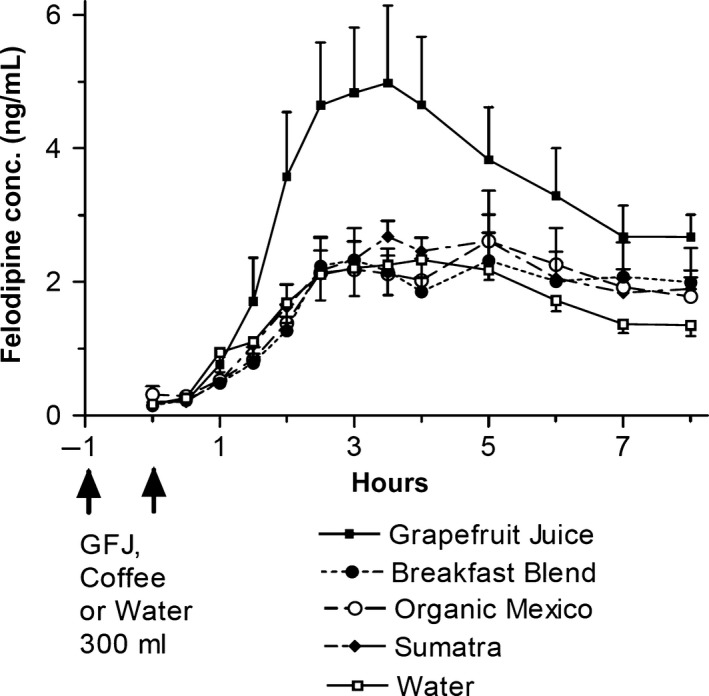
Plasma concentration–time profiles of felodipine with grapefruit juice, coffees and water. Data (*n* = 5) are the mean ± SEM.

Mean plasma felodipine concentration–time profiles for each of the treatments are shown in Figure 2. Increased felodipine AUC and Cmax and decreased dehydrofelodipine/felodipine AUC ratio were observed with grapefruit juice compared to water (Table 1). No differences in pharmacokinetics occurred with the three coffees relative to those with water.

**Table 1:** Felodipine and dehydrofelodipine pharmacokinetics

	Water	Breakfast Blend	Organic Mexico	Sumatra	Grapefruit juice
Felodipine					
AUC_0–8_ (ng.h/mL)	13.0 ± 1.6	13.6 ± 1.9	14.0 ± 1.9	14.6 ± 1.7	25.0 ± 4.3 *P* < 0.001
Cmax (ng/mL)	2.7 ± 0.3	2.7 ± 0.4	3.2 ± 0.7	3.0 ± 0.3	5.8 ± 1.0 *P* < 0.001
Tmax (h)	3.4 ± 0.5	4.2 ± 0.8	3.4 ± 0.7	3.6 ± 0.4	2.9 ± 0.5
t1/2 (h)	4.2 ± 0.9	4.9 ± 0.8	4.1 ± 0.8	5.0 ± 0.7	3.8 ± 0.3
Dehydrofelodipine					
AUC_0–8_ (ng.h/mL)	16.9 ± 2.6	16.9 ± 1.8	18.1 ± 2.2	18.9 ± 1.9	21.0 ± 4.2
Cmax (ng/mL)	4.1 ± 0.7	3.9 ± 0.4	4.6 ± 0.6	4.4 ± 0.4	5.1 ± 0.9
Tmax (h)	3.4 ± 0.5	2.7 ± 0.1	3.3 ± 0.7	3.2 ± 0.3	2.9 ± 0.5
t1/2 (h)	3.3 ± 0.5	3.3 ± 0.6	3.2 ± 0.5	2.7 ± 0.4	3.7 ± 0.6
Dehydrofelodipine/felodipine					
AUC_0–8_ ratio	1.29 ± 0.12	1.27 ± 0.06	1.31 ± 0.09	1.30 ± 0.05	0.84 ± 0.05 *P* < 0.001

Statistically different results are reported for felodipine with water versus felodipine with other treatments.

Individually, felodipine AUC, Cmax, and AUC ratio with grapefruit juice relative to water varied (range: 261%, 275%, 57% to 130%, 107%, 78%, respectively). The greatest individual effect with each coffee versus grapefruit juice compared to water were Breakfast Blend (Subject #4 – 132%, 108%, 78% vs. 198%, 208%, 55%), Organic Mexico (Subject #2 – 120%, 172%, 113% vs. 194%, 275%, 83%) and Sumatra (Subject #2 – 139%, 173%, 107% vs. 194%, 275%, 83%), correspondingly.

#### Caffeine pharmacokinetics

Mean caffeine pharmacokinetics with the coffees are presented in Table 2. Caffeine AUC and Cmax with the Organic Mexico coffee were higher than those with the Breakfast Blend and Sumatra coffees.

**Table 2:** Caffeine pharmacokinetics

	Breakfast Blend	Organic Mexico	Sumatra
Caffeine			
Baseline conc. (μg/mL)	2.8 ± 1.5	2.7 ± 2.3	1.4 ± 1.3
AUC_0–8_ (μg.h/mL)	493 ± 80 *P* < 0.05	627 ± 105	475 ± 54 *P* < 0.05
Cmax (μg/mL)	87 ± 10 *P* < 0.05	116 ± 16	87 ± 12 *P* < 0.05
Tmax (h)	1.7 ± 0.5	1.1 ± 0.2	0.9 ± 0.1
t1/2 (h)	6.2 ± 0.9	7.5 ± 1.2	7.6 ± 1.8

Statistically different results are reported for Organic Mexico coffee versus Breakfast Blend and Sumatra coffees.

## Discussion

Bergamottin is a constituent in grapefruit involved in in vitro and clinical drug interactions and was the positive control for inhibition of CYP3A4 activity in this study for this reason (He et al. 1998; Bailey et al. 2000, 2003; Guo and Yamazoe 2004). It reduced felodipine metabolism less with simultaneous compared to 10‐min prior enzyme exposure. This time‐dependent effect is characteristic of mechanism‐based inhibition, which has the possibility to cause a potent and prolonged effect in vivo. For example, a single 200 mL serving of grapefruit juice markedly enhanced felodipine bioavailability even when this juice was consumed many hours beforehand (Lundahl et al. 1995). Thus, grapefruit and related causative citrus fruits are routinely recommended to be avoided throughout the entire duration of pharmacotherapy with affected drugs (Bailey et al. 2013).

The 1% coffee fractions also demonstrated in vitro mechanism‐based inhibition. The extent of this effect varied among the six coffees. The greatest was with Breakfast Blend (simultaneous minus prior exposure = 8.1 ng/mL, *P* < 0.001) and Organic Mexico (5.6 ng/mL, *P* < 0.001). The least was with Sumatra (3.8 ng/mL, *P* > 0.05). These coffees, respectively, had mild, medium, and extra bold roasting suggesting involvement of this process in the degradation of the active inhibitory constituents. These three coffees underwent clinical testing to determine whether the in vitro effect would be translated to a drug interaction like that with grapefruit.

Grapefruit affected mean felodipine pharmacokinetics as reported previously with higher AUC and Cmax and unaltered apparent elimination half‐life consistent with enhanced oral drug bioavailability (Bailey et al. 1996; Lundahl et al. 1997). Reduced dehydrofelodipine/felodipine AUC ratio indicated decreased CYP3A4 activity (Bailey et al. 1996). The increase in felodipine bioavailability varied among individuals as observed before (Bailey et al. 1995). The likely origin was inherent intestinal CYP3A4 content with a high level resulting in a marked change of pharmacokinetics and vice versa (Lown et al. 1997).

If coffee were to cause in vivo mechanism‐based inhibition of CYP3A4 activity, coffee consumption a number of hours beforehand might mask the true extent of the interaction on the study day. For grapefruit juice, the interaction attenuated with a half‐life of recovery of 12 h, which likely represented the rate of de novo CYP3A4 synthesis, and would be reduced to 12.5% of the maximum effect with an interval of 36 h [3 × CYP3A4 recovery half‐life] (Bailey et al. 1998). Baseline caffeine concentration on the study day was used as a surrogate marker of previous coffee consumption. The average time when it was last consumed was estimated [t1/2 for caffeine × number of t1/2's] between caffeine Cmax and baseline] for Breakfast Blend, Organic Mexico and Sumatra and found to be 30, 37.5, and 46 h ago, respectively. Thus, these intervals seemed sufficient to eliminate any meaningful clinical inhibition of CYP3A4 prior to the study days. This was further supported by the marked interaction observed with the positive control grapefruit juice.

Mean felodipine pharmacokinetics with the three coffees were essentially the same as those with water. Additionally, only one individual receiving Breakfast Blend, the most potent in vitro mechanism‐based inhibitor, had enhanced felodipine AUC and Cmax as well as reduced dehydrofelodipine/felodipine AUC ratio, but these effects were much less than those with grapefruit juice. Consequently, lack of translation of in vitro CYP3A4 inhibitory potency to the clinical circumstance is the most likely interpretation. A similar study also had an analogous outcome. Pomegranate and grapefruit juices had comparable in vitro CYP3A inhibitory potency for triazolam metabolism by human liver microsomes during simultaneous exposure but only grapefruit juice increased oral midazolam Cmax and AUC in healthy volunteers (Farkas et al. 2007). However, pomegranate juice did not cause mechanism‐based inhibition. Thus, our investigation showed that even in vitro mechanism‐based inhibition could not be a reliable predictor of clinical effect.

This failure of translation to the clinical circumstance may reside with inadequate concentration of coffee CYP3A4 inhibitor(s) in the cytoplasm of the small intestinal enterocytes due to issues related to distribution, metabolic inactivation, or disconnect with felodipine absorption. It is possible that more frequent ingestion of coffee during the several hours of felodipine absorption may produce the interaction. Yet, the rationale for conducting this study was based on a previous investigation in which supplementary data showed that four subjects had noticeably elevated felodipine AUC and Cmax with just two servings of coffee (Bailey et al. 2016). Also, two individuals (Subjects #2 and Subject #4 of this study) participated in both investigations. Despite higher felodipine AUC and Cmax (225%, 155% and 165%, 196%, respectively) with coffee initially, these parameters were much less augmented with the three coffees subsequently. In the former case, the coffee came from Columbia, which may be the reason for the discrepancy. However, this requires testing. Regardless, validation of either of the above would likely reduce the practical relevance of this interaction because of the requirement of more frequent coffee consumption or concern for just specific coffees.

Felodipine has essentially complete oral absorption but only an average 15% absolute bioavailability (Edgar et al. 1985). This high presystemic metabolic clearance is preceded by drug delivery to intestines and liver through blood flow. Coffee and caffeine can acutely affect hemodynamics (Nurminen et al. 1999; Riksen et al. 2009; Mesas et al. 2011). Increased systolic and diastolic blood pressure and decreased heart rate are manifested through enhanced arterial resistance and withdrawal of baroreflex‐mediated sympathetic activity, respectively (Pincomb et al. 1985; Casiglia et al. 1991; Notarius et al. 2001; Farag et al. 2005). This might attenuate blood flow to the intestines and liver and alter drug bioavailability characteristics. Although the extent of bioavailability might not be affected, the rate of drug absorption could be reduced. However, felodipine AUC, Cmax, and Tmax with the three coffees were essentially the same as those with water. Vasodilation by felodipine may have been sufficient to overcome the vasoconstriction action by coffee in these organs.

Potential shortcomings of this study might include the small subject sample size. However, it was sufficient to detect a statistically significant effect of grapefruit juice even during intention to treat analysis and thereby was adequate to assess the hypothesis of equivalency of coffee to produce this interaction compared to this fruit juice. Moreover, the pharmacokinetics of felodipine with water and the three coffees were so similar that a power analysis to detect a difference between treatments would not change the conclusion of lack of difference due to inadequate sample size. We studied just three coffees clinically. Yet, they all had a potent in vitro inhibitory effect at low concentration compared to control and two also had a mechanism‐based method of action. Although results may have been opposite than predicted, they provided new information about a potentially common drug pharmacokinetic interaction that had not been previously studied to our knowledge.

In summary, a 1% coffee fraction from six freshly brewed beans from differing geographical location and roasting substantially reduced in vitro CYP3A4 activity. In vitro mechanism‐based inhibition, which appears to be the primary basis for grapefruit–drug interactions, was common. Although grapefruit juice augmented felodipine oral bioavailability, none of the three coffees had this effect. One subject receiving the most potent mechanism‐based inhibitor coffee had an interaction similar to but much less than that with grapefruit. The essential message of this study is that the in vitro active ingredient(s), despite high potency, had essentially negligible action on the clinical pharmacokinetics of felodipine. This result emphasizes the importance of follow‐up clinical testing whenever in vitro results support an interaction. However, this is an infrequent occurrence in the vast literature of just in vitro data.

## Author Contributions

Bailey, Dresser, and Arnold participated in research design. Bailey, Dresser, Urquhart, Proniuk, Tieu, and Freeman conducted experiments.Bailey, Proniuk, and Tieu performed data analysis. Bailey, Dresser, Urquhart, Proniuk, Tieu, and Freeman wrote or contributed to the writing of the manuscript.

## Disclosures

None declared.

## References

[prp2346-bib-0001] Ahnoff M (1984). Determination of felodipine in plasma by capillary gas chromatography with electron capture detection. J. Pharm. Biomed. Anal. 2: 519–526. 16867732 10.1016/0731-7085(84)80055-2

[prp2346-bib-0002] Bailey DG (2010). Fruit juice inhibition of uptake transport: a new type of food‐drug interaction. Br. J. Clin. Pharmacol. 70: 645–655. 21039758 10.1111/j.1365-2125.2010.03722.x PMC2997304

[prp2346-bib-0003] Bailey DG , Spence JD , Edgar B , Bayliff CD , Arnold JMO (1989). Ethanol enhances the hemodynamic effects of felodipine. Clin. Investig. Med. 12: 357–362. 2612087

[prp2346-bib-0004] Bailey DG , Spence JD , Munoz C , Arnold JMO (1991). Interaction of citrus juices with felodipine and nifedipine. Lancet 337: 268–269. 1671113 10.1016/0140-6736(91)90872-m

[prp2346-bib-0005] Bailey DG , Arnold JM , Bend JR , Tran LT , Spence JD (1995). Grapefruit juice‐felodipine interaction: reproducibility and characterization with the extended release drug formulation. Br. J. Clin. Pharmacol. 40: 135–140. 8562295 PMC1365172

[prp2346-bib-0006] Bailey DG , Bend JR , Arnold JM , Tran LT , Spence JD (1996). Erythromycin‐felodipine interaction: magnitude, mechanism, and comparison with grapefruit juice. Clin. Pharmacol. Ther. 60: 25–33. 8689808 10.1016/S0009-9236(96)90163-0

[prp2346-bib-0007] Bailey DG , Malcolm J , Arnold O , Spence JD (1998). Grapefruit juice‐drug interactions. Br. J. Clin. Pharmacol. 46: 101–110. 9723817 10.1046/j.1365-2125.1998.00764.x PMC1873672

[prp2346-bib-0008] Bailey DG , Dresser GK , Kreeft JH , Munoz C , Freeman DJ , Bend JR (2000). Grapefruit‐felodipine interaction: effect of unprocessed fruit and probable active ingredients. Clin. Pharmacol. Ther. 68: 468–477. 11103749 10.1067/mcp.2000.110774

[prp2346-bib-0009] Bailey DG , Dresser GK , Bend JR (2003). Bergamottin, lime juice and red wine as inhibitors of CYP3A4 activity: comparison with grapefruit juice. Clin. Pharmacol. Ther. 73: 529–537. 12811362 10.1016/S0009-9236(03)00051-1

[prp2346-bib-0010] Bailey DG , Dresser G , Arnold JM (2013). Grapefruit‐medication interactions: forbidden fruit or avoidable consequences? CMAJ 185: 309–316. 23184849 10.1503/cmaj.120951 PMC3589309

[prp2346-bib-0011] Bailey DG , Dresser GK , Urquhart BL , Freeman DJ , Arnold JM (2016). Coffee‐antihypertensive drug interaction: a hemodynamic and pharmacokinetic study with felodipine. Am. J. Hypertens. 29: 1386–1393. 27481881 10.1093/ajh/hpw081

[prp2346-bib-0012] Carey RJ , DePalma G (1994). A simplified method for the measurement of caffeine in plasma and brain: evidence for a cortical‐subcortical caffeine concentration differential in brain. J. Neurosci. Methods 53: 19–22. 7990510 10.1016/0165-0270(94)90139-2

[prp2346-bib-0013] Casiglia E , Bongiovì S , Paleari CD , Petucco S , Boni M , Colangeli G , et al. (1991). Haemodynamic effects of coffee and caffeine in normal volunteers: a placebo controlled clinical study. J. Intern. Med. 229: 501–504. 2045756 10.1111/j.1365-2796.1991.tb00385.x

[prp2346-bib-0014] Dresser GK , Bailey DG , Leake BF , Schwarz UI , Dawson PA , Freeman DJ , et al. (2002). Fruit juices inhibit organic anion transporting polypeptide‐mediated drug uptake to decrease the oral availability of fexofenadine. Clin. Pharmacol. Ther. 71: 11–20. 11823753 10.1067/mcp.2002.121152

[prp2346-bib-0015] Edgar B , Regårdh CG , Johnsson G , Johansson L , Lundborg P , Löfberg I , et al. (1985). Felodipine kinetics in healthy man. Clin. Pharmacol. Ther. 38: 205–211. 4017422 10.1038/clpt.1985.160

[prp2346-bib-0016] Farag NH , Vincent AS , McKey BS , Whitsett TL , Lovallo WR (2005). Hemodynamic mechanisms underlying the incomplete tolerance to caffeine's pressor effects. Am. J. Cardiol. 95: 1389–1392. 15904654 10.1016/j.amjcard.2005.01.093

[prp2346-bib-0017] Farkas D , Oleson LE , Zhao Y , Harmatz JS , Zinny MA , Court MH , et al. (2007). Pomegranate juice does not impair clearance of oral or intravenous midazolam, a probe for cytochrome P450‐3A activity: comparison with grapefruit juice. J. Clin. Pharmacol. 47: 286–294. 17322140 10.1177/0091270006298359

[prp2346-bib-0018] Guo LQ , Yamazoe Y (2004). Inhibition of cytochrome P450 by furanocoumarins in grapefruit juice and herbal medicines. Acta Pharmacol. Sin. 25: 129–136. 14769198

[prp2346-bib-0019] Guo LQ , Chen QY , Wang X , Liu YX , Chu XM , Cao XM , et al. (2007). Different roles of pummelo, furanocoumarin and cytochrome P450 3A5*3 polymorphism in the fate and action of felodipine. Curr. Drug Metab. 8: 623–630. 17691921 10.2174/138920007781368917

[prp2346-bib-0020] He K , Iyer KR , Hayes RN , Sinz MW , Woolf TF , Hollenberg PF (1998). Inactivation of cytochrome P450 3A4 by bergamottin, a component in grapefruit juice. Chem. Res. Toxicol. 11: 252–259. 9548795 10.1021/tx970192k

[prp2346-bib-0021] Lown KS , Bailey DG , Fontana RJ , Janardan SK , Adair CH , Fortlage LA , et al. (1997). Grapefruit juice increases felodipine oral availability in humans by decreasing intestinal CYP3A protein expression. J. Clin. Invest. 99: 2545–2553. 9153299 10.1172/JCI119439 PMC508096

[prp2346-bib-0022] Lundahl J , Regårdh CG , Edgar B , Johnsson G (1995). Relationship between time of intake of grapefruit juice and its effect on pharmacokinetics and pharmacodynamics of felodipine in healthy subjects. Eur. J. Clin. Pharmacol. 49: 61–67. 8751023 10.1007/BF00192360

[prp2346-bib-0023] Lundahl J , Regårdh CG , Edgar B , Johnsson G (1997). Effects of grapefruit juice ingestion — pharmacokinetics and haemodynamics of intravenously and orally administered felodipine in healthy men. Eur. J. Clin. Pharmacol. 52: 139–145. 9174684 10.1007/s002280050263

[prp2346-bib-0024] Malhotra S , Bailey DG , Paine MF , Watkins PB (2001). Seville orange juice–felodipine interaction: comparison with dilute grapefruit juice and involvement of the furanocoumarins. Clin. Pharmacol. Ther. 69: 14–23. 11180034 10.1067/mcp.2001.113185

[prp2346-bib-0025] Mesas AE , Leon‐Muñoz LM , Rodriguez‐Artalejo F , Lopez‐Garcia E (2011). The effect of coffee on blood pressure and cardiovascular disease in hypertensive individuals: a systematic review and meta‐analysis. Am. J. Clin. Nutr. 94: 1113–1126. 21880846 10.3945/ajcn.111.016667

[prp2346-bib-0026] Notarius CF , Atchison DJ , Rongen GA , Floras JS (2001). Effect of adenosine receptor blockade with caffeine on sympathetic response to handgrip exercise in heart failure. Am. J. Physiol. Heart Circ. Physiol. 281: H1312–H1318. 11514302 10.1152/ajpheart.2001.281.3.H1312

[prp2346-bib-0027] Nurminen ML , Niittynen L , Korpela R , Vapaatalo H (1999). Coffee, caffeine and blood pressure: a critical review. Eur. J. Clin. Nutr. 53: 831–839. 10556993 10.1038/sj.ejcn.1600899

[prp2346-bib-0028] Pincomb GA , Lovallo WR , Passey RB , Whitsett TL , Silverstein SM , Wilson MF (1985). Effects of caffeine on vascular resistance, cardiac output and myocardial contractility in young men. Am. J. Cardiol. 56: 119–122. 4014015 10.1016/0002-9149(85)90578-8

[prp2346-bib-0029] Riksen NP , Rongen GA , Smits P (2009). Acute and long‐term cardiovascular effects of coffee: implications for coronary heart disease. Pharmacol. Ther. 121: 185–191. 19049813 10.1016/j.pharmthera.2008.10.006

